# Bronze age supply chains between ancient Egypt and Nubia revealed by lead isotope analysis of kohl samples

**DOI:** 10.1038/s41598-024-79162-z

**Published:** 2024-11-11

**Authors:** Rennan Lemos, Matei Tichindelean, Yulia V. Erban Kochergina, Caterina Zaggia, Ludmila Werkström, Emma Hocker, Marcos Martinón-Torres

**Affiliations:** 1 Department of Archaeology and McDonald Institute for Archaeological Research, University of Cambridge, Downing Street, Cambridge, CB2 3DZ UK; 2 Cotsen Institute of Archaeology, University of California, Los Angeles, USA; 3 Department of Rock Geochemistry, Czech Geological Survey, Prague, Czech Republic; 4 Museum Gustavianum, Uppsala University, Uppsala, Sweden

**Keywords:** Kohl, Lead isotope analysis, Supply chains, Sudan, Nubia, Egypt, Geology, Environmental chemistry, Geochemistry

## Abstract

While there is a considerable body of data regarding the sources of minerals employed in Bronze Age Egypt, the supply chains to Sudanese Lower Nubia are virtually unknown. This paper presents results of lead isotope analysis of 11 samples of kohl from C-group, Pan-grave and New Kingdom funerary contexts across the Debeira and Ashkeit areas in Sudanese Lower Nubia during the Bronze Age. The samples could be divided in two groups based on their lead isotope ratios. A comparison between the kohl samples from Sudanese Lower Nubia and galena ores from various mining sites on the Red Sea coast of Egypt indicated that some of the galena used in kohl mixtures in Sudanese Lower Nubia came from the Pharaonic mining site of Gebel el-Zeit. The second group of Nubian samples seems to have originated from an alternative galena source yet to be determined. This is the first time that kohl samples from Sudanese Lower Nubia are integrated into known northeast African networks of supply by using Lead isotope analysis.

## Introduction

Applications of lead isotope analysis (LIA) to a variety of ancient Egyptian materials have demonstrated the validity of this method for determining the provenance of raw materials, shedding light on supply chains and consumption networks^1–9^. This is because the lead isotope ratios of ores coming from different sources of raw materials exploited by ancient settled and mobile communities in northeast Africa are distinctive enough to provide clues regarding their geological origin. While lead isotope ratios of geological samples are sometimes not sufficiently distinctive and may occasionally overlap, thereby complicating the secure determination of the origin of archaeological samples^10^, lead isotope studies of kohl and galena ores from the Egyptian and Sudanese Eastern Desert are well-suited to this approach^2,27^.

**Fig. 1:**
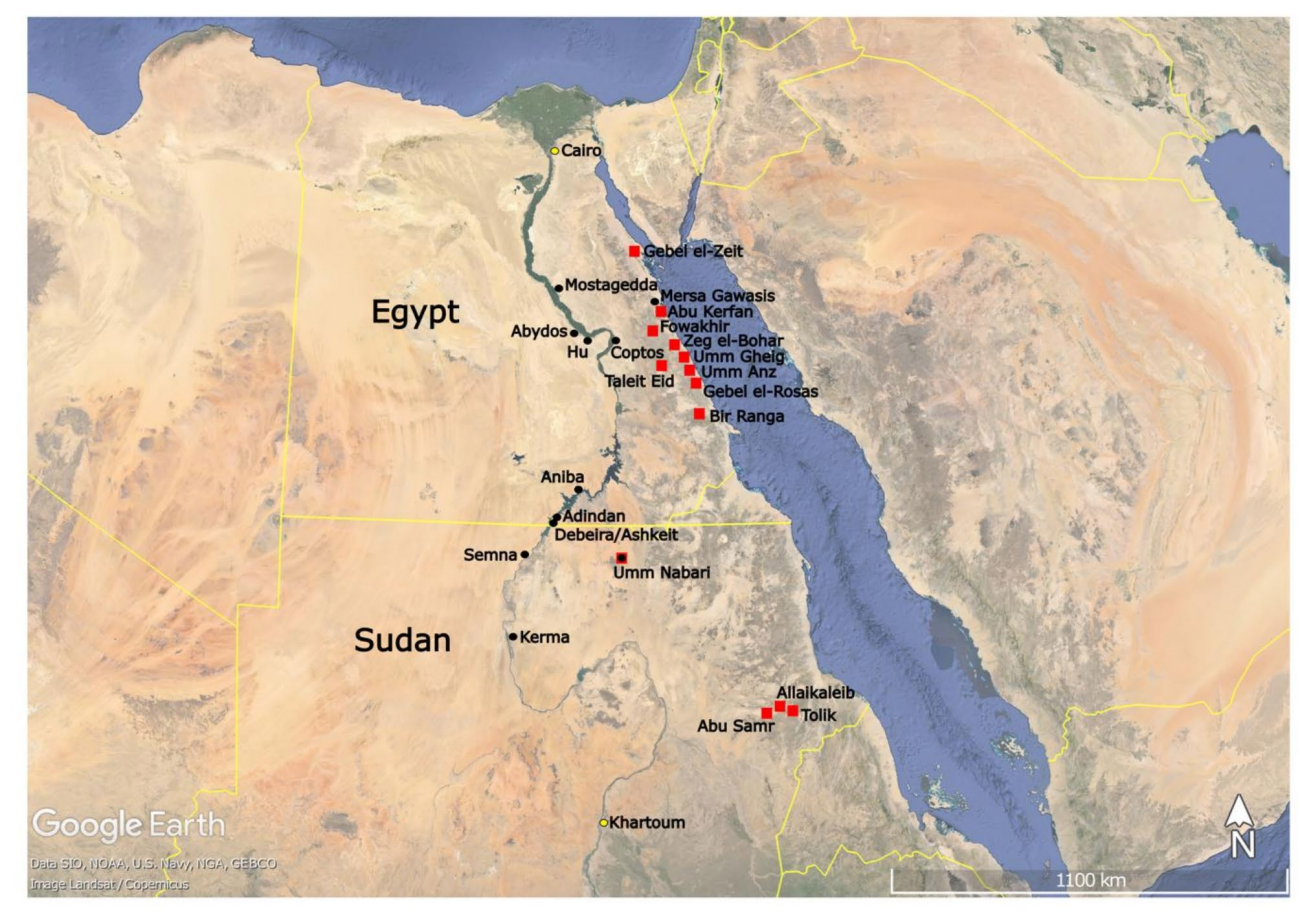
Map of Egypt and Sudan showing the sites mentioned in the text. The red squares represent galena sources, while the black dots indicate archaeological sites. The approximate location of potential galena sources in Sudan are based on Whiteman’s survey^14^. Prepared by R. Lemos using Google Earth Pro.

This paper presents for the first time LIA results of ancient kohl samples from Sudanese Lower Nubia, expanding our knowledge of kohl production and usage in the Nile valley (Fig. 1). LIA is an effective method to analyze these samples, since kohl consists mostly of ground galena mixed with plant- and animal-derived binders^11^. Moreover, several of the challenges to determine the provenance of metal objects by using LIA do not play a major role in the investigation of kohl samples, since the raw mineral does not undergo a pyrotechnological process and is often only mixed with fatty binders. If ores from different sources were mixed, the results obtained would produce intermediate lead isotope ratios^2^. However, given the resulting tight groupings reported here a mixed signature is not likely to have occurred in the bulk powder sampled from the Lower Nubian kohl pots presented in this paper. All samples analyzed for this study originate from ancient Tehkhet, modern Debeira and Ashkeit areas of north Sudan^12^.

Kohls, often applied as body decoration (eyepaints in particular), have been used by various groups in northeast Africa since the Predynastic Period (c. 5000–3100 BCE) into the modern era. The kohl and galena materials covered in this paper are archaeologically dated from c. 2000–1295 BCE, equivalent to the Middle and Late Bronze Age^12^. Kohl containers found in Middle Bronze Age Kerma cemeteries in the third and second cataract areas of the Nile valley likely reached these contexts via diplomacy and trade^15^. The same is probably true for Middle Bronze Age kohl samples from C-group and Pan-grave contexts, which suggests these populations were effectively integrated into Bronze Age networks of exchange within which they interacted with the Egyptian state on a regular basis^16^. The Late Bronze Age kohl samples analyzed in the present study originate from Sudanese Lower Nubian cemeteries^17–19^. During the New Kingdom colonial period, local communities of Sudanese Lower Nubia were impoverished and experienced extreme scarcity, which resulted in various creative strategies to overcome colonial impositions^20–22^. This means that some of the containers used to store kohl substances in this period could have been recycled from earlier contexts to store kohl in the Late Bronze Age.

Galena ores were brought to the Lower Nubian Nile valley from the deserts to be processed locally. For instance, evidence from various workshop sites in the Batn el-Hajar region suggests that kohl was processed in the area, as grindstones and pounders stained with kohl have been reported^23^. While burials in the area are among the poorest in the pharaonic Nubian funerary landscape, a kohl applicator has also been found in a grave^23^. Moreover, the Scandinavian Joint Expedition to Sudanese Nubia (SJE) identified 62 stone vessels that are believed to be kohl containers, a majority of them from the New Kingdom colonial cemetery of Fadrus, located in Debeira East^17^. Additionally, the SJE recovered three kohl pots from Site 47 and three from Site 170 in Debeira East, as well as three kohl pots from Site 95 in Ashkeit (an archaeological site less than 4 km downriver from Debeira East)—all three cemetery sites described as Pan-grave and C-group^24^. It is important to note that these Pan-grave/C-group sites were most likely established before the complete imposition of Egyptian colonial control in the area in the Middle Bronze Age and continued to be used and visited into the early Late Bronze Age or New Kingdom colonial period^17,25^. The Middle Bronze Age cemeteries of Adindan demonstrate that C-group and Pan-grave communities often deposited kohl in bivalve shells or onto palettes during the funerary ritual^26,27^. This indicates that, by the Middle Bronze Age, the use and intentional burial of kohl containers was a widespread, cross-cultural practice in northeast Africa, although more extensively practiced in places controlled by the Egyptian state.

**Fig. 2:**
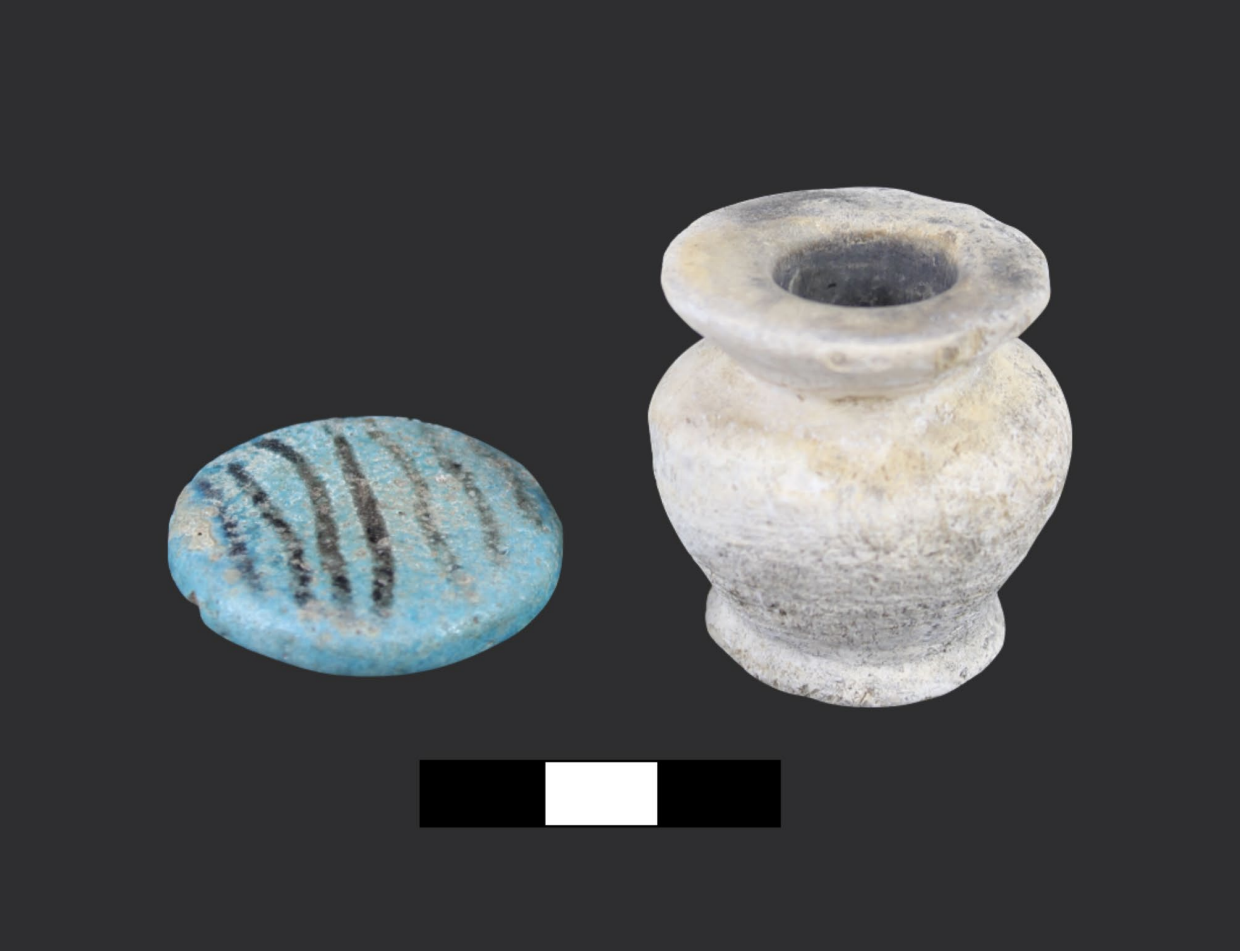
A travertine kohl container with a painted blue faience lid from the cemetery of Fadrus in Debeira (grave 22). Black residue of crushed galena powder can be seen on the inside. Photo by R. Lemos, courtesy of Museum Gustavianum, Uppsala University.

Previous morphological and chemical characterization of kohl in the Nile valley have detected a large diversity of recipes, which might have been connected to different cultural aims^11^. However, the vast majority of analyzed samples remain lead-rich, galena-based mixtures^2,28^. Samples of visible black residue, characteristic of galena-based kohls, were taken from the interior of containers made of several stones (Fig. 2).

The data set explored here consists of kohl containers found in graves in the Debeira and Askheit areas of Sudanese Lower Nubia (Table 1). Nine samples come from the cemetery of Fadrus—the largest non-elite cemetery excavated in Nubia—and date from the 18th Dynasty^17^. The two remaining samples in the corpus date from the Middle Bronze Age; one comes from a C-group cemetery at Ashkeit (Site 183) and the other comes from a Pan-grave cemetery at Debeira East (Site 47)^24^.

**Table 1:** List and contextualization of the analyzed kohl samples from Sudanese Lower Nubia. Data based on^24^ and ^17^.

	Site	Burial: Object	Date	Context
1	Debeira East/Site 185 (Fadrus)	200:7	LBA (New Kingdom)	Side niche burial containing faience beads, scarabs and pottery
2	Debeira East/Site 185 (Fadrus)	22:1	LBA (New Kingdom)	Pit burial containing 4 bronze spiral earrings
3	Debeira East/Site 185 (Fadrus)	56:2	LBA (New Kingdom)	Pit burial (adult) containing a coffin, a scarab and pottery
4	Debeira East/Site 185 (Fadrus)	128:2	LBA (New Kingdom)	Pit burial containing a scarab, a pair of bronze tweezers and pottery
5	Debeira East/Site 185 (Fadrus)	246:10	LBA (New Kingdom)	Mud-brick chamber in pit containing ivory inlay fragments and pottery
6	Debeira East/Site 185 (Fadrus)	248:7	LBA (New Kingdom)	Side niche burial containing a cartonnage coffin, a scarab and pottery
7	Debeira East/Site 185 (Fadrus)	269:1	LBA (New Kingdom)	Side niche burial containing pottery
8	Debeira East/Site 185 (Fadrus)	305:1	LBA (New Kingdom)	Side niche burial containing a grindstone and pottery
9	Debeira East/Site 185 (Fadrus)	512:20 (Burial B)	LBA (New Kingdom)	Mud-brick chamber in pit containing two burials. Burial A had an inscribed stone fragment, a Taweret pendant, silver links, another kohl or ointment jar, a bronze bowl and pottery. Burial B was associated with pottery
10	Ashkeit/Site 183	54:1	MBA (C-group)	Circular pit (adult male + adult female) containing shell beads, faience beads, carnelian beads, a scarab and a kohl stick
11	Debeira East/Site 47	72:1	MBA (Pan-Grave)	Oval pit with tumulus superstructure. Empty, except for broken Middle Kingdom style kohl container

At the present stage, our knowledge of socio-economic aspects, including production and consumption of goods, remains largely limited, especially due to the lack of evidence for workshops at settlement sites in Nubia^29^. Despite the limited archaeological evidence, scientific investigations of pigments, bitumen and metal objects have been shedding light on the production and circulation of such materials^30–36^. For the New Kingdom, sites such as H25 represent a rare opportunity to investigate the circulation of products coming into Nubia from abroad^37^. This paper expands our knowledge of the supply chains that fed Lower Nubian consumption networks in the Middle and Late Bronze Age by exploring the adoption of kohl in Middle and Late Bronze Age Nubia using LIA analysis.

## Results

To avoid methodological problems in establishing the geological provenance of kohl, comparisons were only established between analyzed kohl samples and between those of known galena ores from the Eastern Desert, which provides significant evidence of Egyptian and Nubian activity throughout Antiquity^3,13^. The lead isotopic ratios obtained for the Bronze Age samples from Sudanese Lower Nubia have been grouped with published lead isotopic ratios for ores from Egypt. All published galena ores were considered in comparison with the new kohl ratios from Sudan. Kohl samples from Bronze Age Egypt were also considered in comparison with those from Sudan in order to establish possible consumption networks (Table 2). As with any study based on legacy data, we acknowledge that analytical precision may be different between instruments, but the patterns observed seem sufficiently strong. We illustrate our points with selected plots of lead isotope ratio combinations, but we visually compared all other ratios to ensure that our observations stand.

**Table 2:** Results of lead isotope analysis of Sudanese Lower Nubian kohl samples in comparison with previously published isotopic ratios for relevant Egyptian galena ores and kohl samples.

*N*	Region	Site	Type	Date	^206^Pb/^204^Pb	^207^Pb/^204^Pb	^208^Pb/^204^Pb	^207^Pb/^206^Pb	^208^Pb/^206^Pb	Reference
1	Sudan LN	Site 185	Kohl	NK	19.488	15.660	39.182	0.803	2.010	Reported here for the first time
2	Sudan LN	Site 185	Kohl	NK	19.470	15.651	39.144	0.803	2.010	Reported here for the first time
3	Sudan LN	Site 185	Kohl	NK	18.449	15.596	38.218	0.845	2.071	Reported here for the first time
4	Sudan LN	Site 185	Kohl	NK	19.510	15.656	39.179	0.802	2.008	Reported here for the first time
5	Sudan LN	Site 185	Kohl	NK	18.480	15.602	38.255	0.844	2.070	Reported here for the first time
6	Sudan LN	Site 185	Kohl	NK	18.439	15.599	38.221	0.845	2.073	Reported here for the first time
7	Sudan LN	Site 185	Kohl	NK	18.435	15.595	38.207	0.845	2.072	Reported here for the first time
8	Sudan LN	Site 185	Kohl	NK	18.366	15.602	38.173	0.849	2.078	Reported here for the first time
9	Sudan LN	Site 185	Kohl	NK	18.357	15.591	38.145	0.849	2.078	Reported here for the first time
10	Sudan LN	Site 183	Kohl	CG	19.479	15.654	39.1572	0.803	2.010	Reported here for the first time
11	Sudan LN	Site 47	Kohl	PG	19.386	15.649	39.045	0.807	2.014	Reported here for the first time
12	Egypt	Lahun	Kohl	NK	19.506	15.685	39.246	0.80409	2.012	[38]
13	Egypt	Lahun	Kohl	NK	19.483	15.657	39.152	0.80366	2.0096	[38]
14	Egypt	?Abydos	Kohl	NK	19.452	15.629	39.100	0.80346	2.0101	[38]
15	Egypt	?Abydos	Kohl	NK	19.450	15.632	39.111	0.80369	2.0108	[38]
16	Egypt	?Abydos	Kohl	NK	19.469	15.651	39.155	0.80388	2.0111	[38]
17	Egypt	Abydos	Kohl	NK	19.415	15.647	39.093	0.80591	2.0135	[38]
18	Egypt	Abydos	Kohl	NK	19.466	15.658	39.136	0.8044	2.0105	[2]
19	Egypt	Abydos	Kohl	NK	19.52	15.700	39.302	0.8043	2.0134	[2]
21	Egypt	Abydos	Kohl	NK	19.361	15.638	39.010	0.8077	2.0149	[2]
22	Egypt	Abydos	Kohl	NK	19.478	15.660	39.143	0.804	2.0096	[2]
23	Egypt	Abydos	Kohl	NK	19.76	15.689	39.399	0.794	1.9939	[2]
24	Egypt	Abydos	Kohl	NK	19.89	15.679	39.446	0.7883	1.9832	[2]
25	Egypt	Abydos	Kohl	NK	19.468	15.644	39.096	0.8036	2.0082	[2]
26	Egypt	Abydos	Kohl	NK	19.464	15.661	39.136	0.8046	2.0107	[2]
27	Egypt	Abydos	Kohl	NK	19.473	15.654	39.131	0.8039	2.0095	[2]
28	Egypt	Abydos	Kohl	NK	19.867	15.707	39.510	0.7906	1.9887	[2]
29	Egypt	Hu	Kohl	PG	19.655	15.671	39.279	0.797	1.998	[2]
30	Egypt	Hu	Kohl	PG	19.207	15.623	38.852	0.813	2.022	[2]
31	Egypt	Hu	Kohl	PG	18.447	15.638	38.385	0.847	2.080	[2]
32	Egypt	Mostagedda	Kohl	PG	19.443	15.644	39.077	0.804	2.009	[2]
33	Egypt	Bir Ranga	Ore	-	18.595	15.601	38.418	0.839	2.066	[39] [40] [41]
34	Egypt	Bir Ranga	Ore	-	18.574	15.539	38.246	0.837	2.059	[39] [41]
35	Egypt	Bir Ranga	Ore	-	18.595	15.589	38.405	0.838	2.065	[42] [40]
36	Egypt	Gebel el-Rosas	Ore	-	19.000	15.580	38.613	0.820	2.032	[39] [40] [41]
37	Egypt	Umm Anz	Ore	-	19.037	15.608	38.741	0.820	2.035	[42]
38	Egypt	Umm Anz	Ore	-	19.090	15.681	38.921	0.821	2.039	[43] [40]
39	Egypt	Umm Anz	Ore	-	19.006	15.579	38.623	0.820	2.032	[39] [41]
40	Egypt	Taleit Eid	Ore	-	20.754	15.693	41.003	0.756	1.976	[39] [41]
41	Egypt	Taleit Eid	Ore	-	20.755	15.694	41.005	0.756	1.976	[42] [43] [40]
42	Egypt	Umm Gheig	Ore	-	19.155	15.626	38.699	0.816	2.020	[42]
43	Egypt	Umm Gheig	Ore	-	19.155	15.625	38.699	0.816	2.020	[43] [40]
44	Egypt	Umm Gheig	Ore	-	19.087	15.594	38.563	0.817	2.020	[44] [40]
45	Egypt	Umm Gheig	Ore	-	19.101	15.600	38.606	0.817	2.021	[38]
46	Egypt	Umm Gheig	Ore	-	19.096	15.607	38.590	0.817	2.021	[45]
47	Egypt	Umm Gheig	Ore	-	19.153	15.628	38.690	0.816	2.020	[39] [41]
48	Egypt	Umm Gheig	Ore	-	19.087	15.595	38.564	0.817	2.020	[39] [41]
49	Egypt	Umm Gheig	Ore	-	19.096	15.602	38.568	0.817	2.020	[39] [41]
50	Egypt	Umm Gheig	Ore	-	19.069	15.592	38.515	0.818	2.020	[39] [41]
51	Egypt	Umm Gheig	Ore	-	19.155	15.626	38.699	0.816	2.020	[39] [41]
52	Egypt	Zeg el-Bohar	Ore	-	18.937	15.585	38.384	0.823	2.027	[45]
53	Egypt	Fowakhir	Ore	-	17.826	15.513	37.381	0.870	2.097	[42]
54	Egypt	Fowakhir	Ore	-	17.830	15.500	37.349	0.869	2.095	[43] [40]
55	Egypt	Abu Kerfan	Ore	-	19.621	15.642	39.060	0.797	1.991	[45]
56	Egypt	Gebel el-Zeit	Ore	-	19.700	15.789	39.636	0.802	2.012	[46] [41]
57	Egypt	Gebel el-Zeit	Ore	-	19.370	15.630	39.035	0.807	2.015	[45]
58	Egypt	Gebel el-Zeit	Ore	-	19.427	15.651	39.115	0.806	2.013	[45]
59	Egypt	Gebel el-Zeit	Ore	-	20.012	15.716	39.682	0.785	1.983	[45]
60	Egypt	Gebel el-Zeit	Ore	-	19.497	15.630	39.092	0.802	2.005	[45]
61	Egypt LN	Adindan	Kohl	CG	18.464	15.574	37.790	0.844	2.047	[27]
62	Egypt LN	Adindan	Kohl	CG	18.433	15.550	37.711	0.844	2.046	[27]
63	Egypt LN	Adindan	Kohl	CG	18.454	15.566	37.763	0.844	2.046	[27]
64	Egypt LN	Adindan	Kohl	CG	18.443	15.561	37.748	0.844	2.047	[27]

**Fig. 3:**
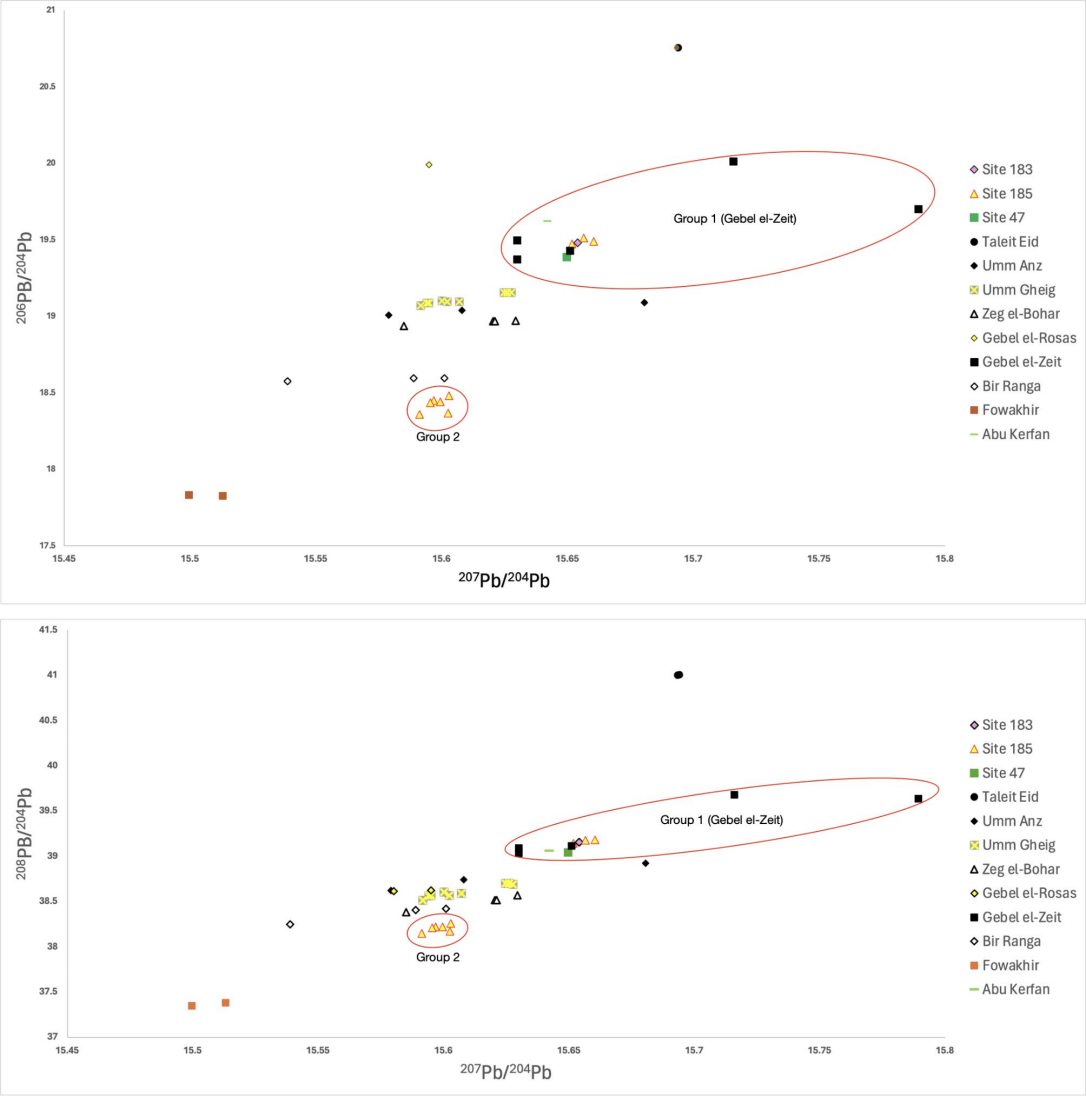
Comparison of lead isotopic ratios of kohl samples from Sudanese Lower Nubia and galena ores from various Egyptian Eastern Desert mining sites along the Red Sea coast. All kohl samples from Sudanese Lower Nubia date from the Middle Bronze Age (C-group and Pan-grave) and the Late Bronze Age (New Kingdom). Raw data compiled in Table 2.

Two main clusters become apparent by comparing lead isotopic ratios of kohl samples from Sudanese Lower Nubia, and galena ores and kohl samples from Egypt. The samples in the first cluster have a strong association with the lead isotopic ratios of ores from the Gebel el-Zeit mining region and originate from Fadrus (Site 185) (samples 1, 2 and 4), Site 183 (sample 10), and Site 47 (sample 11). While kohl containers are far less prevalent in non-Egyptian contexts, like Site 183 (one kohl container reported) and Site 47 (three kohl containers reported), the obtained results indicate a close association of the two sites with Egyptian kohl extracted from Gebel el-Zeit. The second cluster comprises samples from Fadrus only (samples 3, 5, 6, 7, 8 and 9). Based on the available comparative data, it remains difficult to determine their provenance with certainty (Fig. 3). Interestingly, a clear diversity is observed in kohl samples belonging to Nubian groups, such as the Pan-grave, yet their choice of sources frequently lie outside the ones exploited by the ancient Egyptians^2^.

The first group’s lead isotopic ratios cluster together very tightly, strongly indicating that they derive from a single source, which is consistent with the lead isotopic ratios of ores from the Gebel el-Zeit deposits (Fig. 3). While the accuracy of previously published lead isotopic ratios may vary, we note that all of our samples fall in the relatively large isotopic field demarcated by Gebel el-Zeit, including a perfect match between one geological specimen and our archaeological group. This allows us to strongly suggest a geological origin for the analyzed samples. The inclusion of a geological sample from Abu Kerfan in this group only demonstrates the wider variability of isotopic ratios in the Gebel el-Zeit region. Galena from Abu Kerfan occurs as intrusions in limestone deposits and should probably be considered in the context of ores from the Gebel el-Zeit mining region^45^.

All the samples in group 2 have lead isotopic ratios that are in close association with each other, forming another coherent cluster. This likely indicates their common origin; even if reuse of containers for kohls from different sources might result in samples with mixed signatures, the similarity within this group renders that possibility unlikely. These samples plot in relatively proximity with ores from Bir Ranga. However, since the overlap is not perfect, we cannot discount the possibility of a yet unknown source. Perhaps a Sudanese galena source might offer an alternative to Bir Ranga, especially in light of galena deposits in the Sudanese Eastern Desert and along the Red Sea coast^14^. In the absence of published ore lead isotopic ratios for Sudan, such an assumption remains tentative at the current stage. Nevertheless, it is evident that the people of Fadrus had access to at least two different ore sources, one of which most likely being Gebel el-Zeit.

## Discussion

Based on the above, the majority of galena-based kohl mixtures analyzed in this study dating from the Middle and Late Bronze Age in Sudanese Lower Nubia have strong isotopic affinities with ores from Gebel el-Zeit. While Bir Ranga is a potential source for the second cluster, we cannot rule out an as yet uncharacterized Eastern/Nubian Desert source. The extensive mines of Gebel el-Zeit were one of the main sources of galena for the Egyptian state during the second millennium BCE. Seasonal encampments and other installations dating to as early as the Middle Kingdom and continuing into the New Kingdom have also been detected in association with shaft mines, which provide a chronological timeframe for ancient mining activities at the site^47–49^. The archaeological evidence for Bronze Age exploitation of the Bir Ranga deposit remains scant. While the mines at Bir Ranga, Umm Gheig, and Gebel el-Rosas have pre-modern evidence of mining activities, the dearth of published archaeological materials does not allow us to draw concrete conclusions^2,45^.

Even so, the LIA results presented in this study illuminate two potential sources of procurement of galena accessible to Bronze Age communities in Sudanese Lower Nubia. Most interestingly, it suggests that the local C-group and Pan-grave populations had access to materials originating from the Gebel el-Zeit mines controlled by the Egyptian state and made available to colonized communities in New Kingdom Nubia^47,48^. In particular, the samples from Site 183 (C-group) and Site 47 (Pan-grave) cluster together with samples from Egyptian pre-Hatshepsut (c. 1473–1458 BCE) burials, suggesting a close relationship between these communities and the ones in Debeira East/Ashkeit during the early 18th Dynasty^17^. This adds depth to current discussions on different modes of social organization coexisting and interacting in northeast Africa^50–54^.

**Fig. 4:**
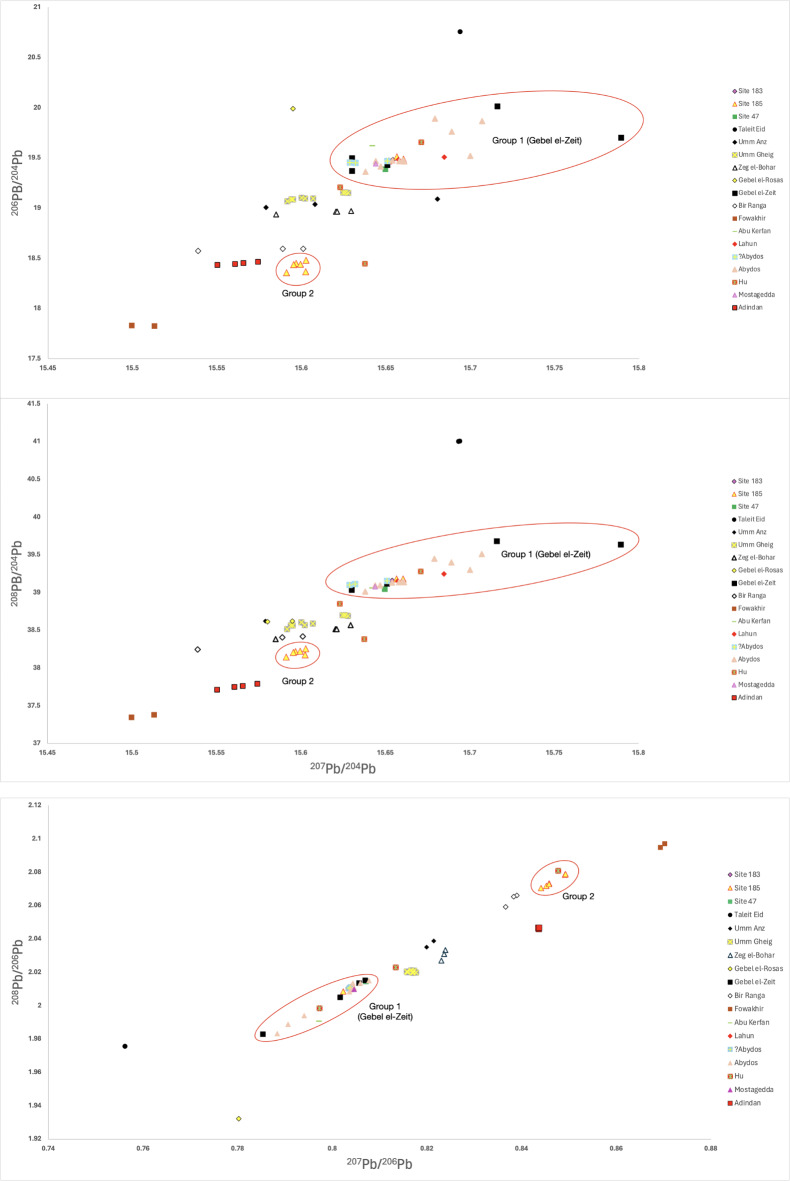
Comparison of ^208^Pb/^206^Pb, ^207^Pb/^206^Pb, ^208^Pb/^204^Pb, ^207^Pb/^204^Pb and ^206^Pb/^204^Pb isotope ratios of Bronze Age kohl samples from Sudanese Lower Nubia and Egypt, and galena ores from various Egyptian Eastern Desert mining sites along the Red Sea coast. Raw data compiled in Table 2.

C-group and Pan-grave pastoralist communities in Sudanese Lower Nubia most likely had access to Egyptian state-controlled chains of supply as a result of commercial relations. A similar cooperation scenario can be proposed for two of the previously analyzed Pan-grave kohl samples, one from Hu and the other from Mostagedda^2,38^. The Pan-grave cemeteries at Hu and Mostagedda are firmly located within ancient Egyptian communities which would have ostensibly necessitated a multi-partisan set of negotiations resulting in a place demarcated and reserved for Pan-grave burials in an overall Egyptian context. As these burials show both cultural and economic sharing of raw and finished material products, as well as diversity in the acquisition or access to galena sources^2^, we propose that nomadic, Pan-grave communities operated as independent, as well as, attached agents in a supra-regional exchange network, therefore granting access to state-controlled supply chains to communities who would otherwise not be able to access circulating materials. The collection of Pan-grave (and C-group) samples plotted in Fig. 4 reflects a probable relation to the nomadic character of Pan-grave communities. On the one hand, the regular and highly mobile nature of the Pan-grave communities indicates diverse access to unknown sources, particularly those outside or different from the ones under the control of large-scale state exploitation. On the other, cooperational endeavors between Egyptian and Pan-grave nomadic communities are also strongly suggested by the ability to access the same networks of galena extraction focusing at the mines of Gebel el-Zeit^2,38^ (Fig. 4).

The overlap, in group 1, of samples belonging to mobile Pan-grave communities, settled colonized Nubian communities in Debeira and Egyptian communities living under the control of the state, is consistent with the proposition that these groups cooperated in the extraction and distribution of galena from the Egyptian and potentially Sudanese Eastern Desert. Such interactions also appear, in a more limited way, in ancient Egyptian textual sources attesting to trade relations between Egyptians and Nubians in occupied Lower Nubia during the Middle Kingdom^55^. It appears that the majority of the kohl present in Egyptian contexts was likely obtained through state-led initiatives and intermediaries that concentrated their efforts on the extensive deposits at Gebel el-Zeit. The site is relatively close to Egyptian settlements in the Nile valley and their large-scale exploitation is evident not only in the large mine galleries but also in the duration of exploitation at various sites in the region^47,48,56^. While Site 47 at Debeira East, a Pan-grave cemetery, was established in close proximity to Egyptian fortresses and settlements in the area, it appears that cooperation and trade between the two communities resulted not only in shared practices but also shared supply chains. The same applies to pastoralist C-group communities living in Sudanese Lower Nubia at Site 183. However, the C-group community at Adindan was clearly attached to a different supply network. Their kohl samples group closely with each other, yet they do not appear to be in relationship with any known sources (Fig. 4). Considered together, the Adindan cluster and our group 2 might suggest the existence of alternative networks of resource extraction coexisting with mainstream, Egyptian state-sponsored networks.

Regarding the provenance of the second group of samples, archaeological and textual evidence might suggest a local origin in Sudanese Lower Nubia for some of the kohl used at Fadrus. As the majority of the samples in group 2 (samples 6, 7, 8, and 9) are dated to the reign of Hatshepsut and her successor, Thutmose III, we propose that a shift in galena-procuring networks occurred at Debeira East/Ashkeit sometime around the second half of the 18th Dynasty^17^. Perhaps this shift was a result of obsolete networks of exchange, as the abundance of C-group and, for the most part, Pan-grave assemblages began to decline in this part of Lower Nubia around the beginning of the 18th Dynasty^25^. Conversely, the desire for new or closer galena sources was an initiative proposed by local leaders sometime around the reign of Hatshepsut.

Textual evidence from Umm Nabari in the Sudanese Eastern Desert attests to the direct involvement of Lower Nubian chiefs from Debeira and Miam/Aniba, such as Djehutyhotep (reign of Hatshepsut) and Hekanefer (reign of Tutankhamun, c. 1336–1327 BCE) respectively, in gold mining expeditions to the Nubian desert in the New Kingdom^57,58^. If one presupposes those desert resource extraction expeditions paralleled Egyptian endeavors into the Egyptian Eastern Desert, then expeditionary forces under Djehutyhotep’s or Hekanefer’s supervision would have sought to collect a diverse selection of resources in order to increase gains from their initial investment. Such strategies are attested in New Kingdom textual records for expeditionary forces heading into the Wadi Hammamat, a way that connected Coptos to the Red Sea coast, and the hinterlands, where both gold and galena were extracted^59^. This strategy is also documented archaeologically, as suggested by Castel and Pouit who detailed Egyptian simultaneous exploitation of gold, copper, and galena sources in the Wadi Dara basin near Gebel el-Zeit^56^. Despite the absence of archaeological evidence for ancient galena exploitation in the Sudanese deserts, the occurrence of small quantities of galena alongside gold-bearing quartz veins has been reported in the Umm Nabari area, which is significant in light of the available textual evidence^14^. Furthermore, Whiteman lists three additional geological sources of galena in the Sudanese Eastern Desert at Tolik, Abu Samr, and Allaikaleib^14^.

Alternatively, galena ore could have arrived at Fadrus through local intermediaries, such as highly mobile Pan-grave and Jebel Mokram groups. While the mobility patterns of the Jebel Mokram group are still emerging, the group’s ceramic tradition is closely associated with other Nubian groups, so much so that a direct link between Pan-grave and Jebel Mokram groups has been proposed^25,60^. Similarly, Pan-grave ceramic evidence from Mersa Gawasis attests to the group’s north-south mobility^61^. This is not only supported by Pan-grave ceramic material as far north as the Delta region in Egypt^25^ but also by the results of our LIA, which places kohl residue from Site 47 in close association with Gebel el-Zeit ore deposits. The temporary Egyptian harbor of Mersa Gawasis is located about 150 km south of Gebel el-Zeit and in relative closer proximity to the Bir Ranga deposits. Therefore, it is plausible to suggest that Nubian communities had direct or indirect knowledge of Gebel el-Zeit and other Egyptian/Sudanese sources of galena. The nomadic Pan-grave group’s knowledge and access to independent and Egyptian galena networks is further evidenced by the diverse lead isotope ratios present in our study and previous reports^2^. While the lead isotopic ratios of samples from Site 47, Hu, and Mostagedda are associated with galena ores from Gebel el-Zeit, another sample from Hu represents an outlier that most likely represents an as yet unknown source^2^.

## Conclusion and future steps

The application of lead isotope analysis to kohl samples from Sudanese Lower Nubia has contributed to expanding our knowledge of supply chains that fed consumption networks in Nubia in the Middle and Late Bronze Age. Samples from C-group, Pan-grave and New Kingdom contexts in Sudanese Lower Nubia clustered together with Egyptian samples dating from the same periods alongside ores from the Gebel el-Zeit galena mines. This suggests that there was a direct connection between the state-led exploitation of galena in Egypt and consumption of kohl in Sudanese Lower Nubia before and during the Egyptian colonization of the area by both settled and mobile groups.

Alternative groupings of Nubian kohl samples further suggest that other, likely local sources of galena were connected to the consumption of kohl in Sudanese and Egyptian Lower Nubia. However, given the current unavailability of ore data for Sudan and the limited amount of information for Egypt, it remains impossible to determine the provenance of the rest of the kohl samples used in Sudanese Lower Nubia.

Future research into Nubian archaeometallurgy will find the results presented here useful, especially in the absence of ore data for Sudan. Recent research into Nubian metallurgy has been producing new lead isotopic data, including information for key sites such as Aniba, Amara West and Kerma^7,35,36^. Comparing these LI ratios with kohl data could shed light onto whether ores from the same sources were used in kohl production and metallurgy. The methodological challenges of such a comparison remained outside the scope of the present contribution.

## Methods

Lead isotopes were measured by a Multiple Collector Inductively Coupled Plasma Mass Spectrometer (Thermo Fisher Scientific Neptune MC-ICP-MS). Prior to undertaking each analysis, the instrument was stabilized for at least 5 h and all parameter setups were optimized daily. Samples were introduced into plasma using a spray chamber and the MC-ICP-MS was operated in the low-resolution mode. Samples for Lead isotope analysis were spiked with thallium (Tl) NIST SRM 997 and mass bias was corrected with the generalized power law using ^205^Tl/^203^Tl = 2.3871. Details of the analytical procedure, along with long-term repeatability and inter-laboratory reproducibility can be found elsewhere^62^. The presented data was uncertainty-weighted by means of three or more replicate measurements. Standard–sample bracketing was used to correct isotopic data relative to NIST SRM 981 (Table 3)^63^.

**Table 3:** Used reference values for NIST 981.

	206Pb/204Pb	207Pb/204Pb	208Pb/204Pb	207Pb/206Pb	208Pb/206Pb
Average	16.9433	15.5008	36.7294	0.9149	2.1678
2SD	0.0307	0.0305	0.1066	0.0002	0.0024

In order to narrow down the provenance of analyzed samples, ^208^Pb/^206^Pb, ^207^Pb/^206^Pb, ^208^Pb/^204^Pb, ^207^Pb/^204^Pb and ^206^Pb/^204^Pb ratios for all kohl samples from Sudanese Lower Nubia were compared with previously analyzed galena ores from known mining sites along the Red Sea coast of Egypt and kohl samples from various Egyptian sites. The comparison aimed to generate clusters that could be subsequently associated with specific regions.

## Data Availability

All data generated or analysed during this study are included in this published article.
